# At the boundary: Law and AI

**DOI:** 10.1073/pnas.2606704123

**Published:** 2026-07-20

**Authors:** Daniel E. Ho, Julian Nyarko, Vanessa Parli, Christopher D. Manning

**Affiliations:** ^a^ Stanford Law School, Stanford University, Stanford, CA 94305; ^b^ Department of Political Science, Stanford University, Stanford, CA 94305; ^c^ Department of Computer Science, Stanford University, Stanford, CA 94305; ^d^ Stanford Institute for Human-Centered Artificial Intelligence (HAI), Stanford University, Stanford, CA 94305; ^e^ Stanford Institute for Economic Policy Research (SIEPR), Stanford University, Stanford, CA 94305; ^f^ Regulation, Evaluation, and Governance Lab (RegLab), Stanford University, Stanford, CA 94305; ^g^ Legal Innovation through Frontier Technology Lab (liftlab), Stanford University, Stanford, CA 94305; ^h^ Department of Linguistics, Stanford University, Stanford, CA 94305

At a recent judicial conference, Justice Elena Kagan offered her thoughts on AI: “Claude, I thought, did an exceptional job of figuring out an extremely difficult Confrontation Clause issue,” she reflected. “On the one hand I thought that was refreshingly humble . . . But then on the other hand it just seems ridiculous that Claude could do an argument better than . . . I could. I kind of think I’m better than Claude” (1).

Yet another Supreme Court Justice, Chief Justice Roberts, was more cautious. In his state-of-the-judiciary report, he flagged that AI could risk “dehumanizing” the law, noting in particular the serious potential for hallucinations (2). While Justice Kagan highlighted a compelling example of Claude analyzing the Confrontation Clause, the legal profession has also seen numerous cases in which AI systems generated inaccurate or entirely fabricated legal authorities (3). By one count, hallucinations have arisen in over 1,600 cases (4). And such hallucinations can have significant consequences. The *New York Times* reported on one case involving a district attorney’s office using AI to keep individuals in jail, with filings allegedly chock full of hallucinations, when the public defender’s office lacked access to such tools (5).

One of the greatest hopes for AI is that it can help to address the acute crisis in access to justice. The United States has over 1.3M lawyers, one of the highest attorney per capita rates in the world. Yet it ranks 112th out of 143 countries in terms of “accessibility and affordability of civil justice” (6). This justice gap is most acute for the most vulnerable subpopulations. As the Legal Services Corporation reports, “Low-income Americans do not get any or enough legal help for 92 percent of their substantial civil legal problems” (7). Yet if prosecutors have access to legal AI and defenders do not, this narrative may turn out to be mistaken. Instead of addressing the access to justice crisis, AI may deepen it.

How should we think about the field of law and AI? What is the appropriate role of AI in legal practice? What can law offer to manage and govern technology? We are pleased to introduce this Special Feature, “Law in the Age of Generative AI,” that tries to move beyond the headlines and understand the appropriate role of AI in law and the role that law has in helping to govern technology. We are excited to convene a group of lawyers, computer scientists, and social scientists to tackle these issues.

## Law as AI Governance

The eight papers in this feature cover a lot of terrain. One set of papers examines the potential for law to help govern technology.

Samuelson (8) discusses the one area of law that could “bring generative AI to its knees:” copyright. It has been fiercely contested whether the training of foundation models violates the rights of copyright holders. Foundation models gain their power because of the sheer size of the training dataset, but does such ingestion violate “fair use?” In December 2023, the New York Times filed a lawsuit against OpenAI and Microsoft for unauthorized use of its articles in training AI models. In the same year, Google was sued for “secretly stealing everything ever created and shared on the internet by hundreds of millions of Americans” and using these data to train its AI. Samuelson examines the arguments and potential for collective licensing solutions, such as through statutory remuneration or commercialization levies. Samuelson highlights the prohibitive transaction costs and the complex normative, economic, and practical problems that licensing would need to address.

Laufer et al. (9) take a step back to study the incentive structure of AI safety when there are foundation model developers (e.g., Anthropic, OpenAI) and downstream deployers who adapt such models for specific applications. A central question in AI governance has been whether it is more effective to impose obligations on developers or deployers. The former have much more capacity to intervene with data and model development, while the latter know much more about the specific deployment risks. Laufer et al. argue that “weak regulations targeting domain specialists alone can unintentionally reduce safety” and that stronger regulations applied to both developers and deployers can act as a commitment device, leading to improvements both on performance and safety dimensions. The piece hence offers a rigorous framework to understand incentives posed by AI regulatory proposals.

Hadfield (10) tackles the question of regulatory and legal infrastructure. Instead of asking, as Laufer et al. do, on whom a substantive obligation should be placed, Hadfield considers proposals for how government can develop the legal infrastructure for imposing substantive rules. Hadfield argues that registration of frontier foundation models, registration of AI agents, and regulatory markets (i.e., licensed private providers who develop technology to ensure that AI systems comply with a government safety standard) will enhance the legibility of AI systems to public authorities.

Last, Chouldechova and Hemel (11) engage in an in-depth analysis of the Supreme Court’s decision in Students for Fair Admissions v. President and Fellows of Harvard College (SFFA), which found college admissions on the basis of race violate equal protection. Using the rise of machine learning in college admissions as a motivating example, they distinguish between “first-order” and “second-order” race consciousness in the training and inference stages of machine learning. By first-order race consciousness, they mean the direct utilization of race as a feature; and second-order race consciousness means that a developer is conscious in the design of a system, without directly using a race attribute. They argue that SFFA may permit—and possibly endorse—some forms of race consciousness. This contribution is an attempt to translate how the Supreme Court equal protection doctrine applies to specific design decisions undertaken in the machine learning process to potentially address a principal concern of algorithmic bias.

## AI in Law

The other set of papers examines how AI can be used to understand, apply, or refine the use of AI in law.

Jung et al. (12) are motivated by the same concern of algorithmic fairness as Chouldechova and Hemel (11), but suggest an alternative to common methods of assessing disparate impact. They offer a risk-adjusted regression framework, to a) estimate risk, b) assess disparities conditional on risk, and c) conduct a sensitivity analysis of estimates. They apply this framework to a stop-and-frisk dataset in New York City and find that the proposed approach reveals sharper disparities than conventional approaches. These methodological refinements may be central in disparate impact and Jung et al. provocatively ask whether policymakers should be “obliged—legally or ethically—to adopt them.”

Ash et al. (13) demonstrate the potential for improved citation network analysis with a corpus of all U.S. federal opinions from 1790 to 2024. Advances in computing have enabled such large-scale analyses and Ash et al. show that unsupervised learning on this network, blinded to courts and topics, recovers the structure of judicial hierarchy and topics. They argue that this points to “the federal common law as a self-organizing network.”

He et al. (14) study how legal interpretation can potentially aid in efforts by AI developers to “align” systems with human values. The notion of “constitutional AI,” for instance, aims to align AI system outputs with specified “constitutional” values, specified in natural language (15). He et al. address the fact that even when values are specified, the law teaches us that interpretive ambiguity remains. They propose a computational framework for rule refinement that minimizes interpretive disagreement by revising ambiguous rules and prompt-based interpretive constraints to improve reliability in rule application.

Guha et al. (16) address the challenge that while legal AI tools are rapidly springing up, there is an underprovision of systematic information about such systems. In contrast to AI systems generally, legal AI lacks “legibility,” as conventional artifacts such as data and model documentation and benchmarking are unavailable, impeding responsible adoption. They adopt an institutional approach and argue that without attending to the challenges of institutional design, benchmarks can be “captured, watered down, and abused.” They propose interventions that match available resources to improve the ecosystem for legal AI innovation.

## Unifying Themes

While the papers cover a lot of terrain both methodologically (statistics, network analysis, large language models) and substantively (algorithmic fairness, AI safety, intellectual property), they also yield productive connections and themes.

First, AI poses a “legibility” problem for both the state and the profession. Hadfield (10) argues that the state currently does not have visibility into frontier models and AI agents, which motivates a registration regime. Guha et al. (16) invoke this concept discussing the legal profession itself, arguing that commercial legal AI tools are “illegible” to practitioners, as they lack public information about design, risk assessments, and benchmarks. The only evaluation measure that a major New York law firm could develop after a year and a half of testing an AI tool, for instance, was “how much lawyers like using it” (17). This lack of legibility impedes both regulatory and professional oversight.

Second, several of the papers borrow from legal institutions to address more technical AI problems. He et al. (14) analogize their framework to administrative rulemaking and adjudication, which iteratively give precision to broad and more vague statutory frameworks. Chouldechova and Hemel’s (11) efforts to translate the Supreme Court’s SFFA decision into a technical guide also attempts to identify lawful mechanisms to ensure algorithmic fairness.

Third, several authors address the allocation of responsibility across general model developers and downstream deployers. Laufer et al. (9) show that if safety regulation only targets deployers, developers will free-ride and lower safety investments. Samuelson (8) observes that large publishers (upstream) may successfully negotiate training data licenses, but individual authors (downstream) face substantial transaction costs, making collective licensing infeasible without some intervention. Hadfield’s (10) solution can be conceived of as proposing intermediaries that act as a bridge, translating government performance goals into technical requirements for developers.

Fourth, several papers are engaged in a dialogue around how to dissect bias in existing systems. Jung et al. (12) critique conventional regression analysis that can mask discrimination by including variables that are correlated with race but unrelated to the actual risk. Chouldechova and Hemel (11) articulate what they call the paradox of second-order race consciousness. The decision of which variables to include itself has distributive consequences. Actively curating these can generate the very cross-racial resentment the Supreme Court seeks to avoid; but omitting them may just as well generate harms.

Last, these papers offer different perspectives of law as an adaptive system. Ash et al. (13) argue that the federal common law maintains stability through “doctrinal backbones,” while allowing for “mutations” in response to social change. In Hadfield’s (10) terms, this raises the question of how the law can adapt to the rise of generative and agentic AI to preserve its backbone while evolving legal infrastructure.

## Open Questions Toward Responsible Legal AI

The field of law and AI is at a particularly exciting time. As these papers illustrate, the capacity to analyze large volumes of legal data—sifting through millions of deed records (18), contracts (19), and cases (20)—is evolving at a remarkable pace. At the same time, core questions of legal reasoning and value alignment remain serious challenges. How should we train models when law is contested? How can rule refinement be achieved without resorting to merely consistency? What are the better ways in which we can evaluate the lawyer–AI interaction? What these papers most acutely demonstrate is the tremendous synergy of working at the boundary of these fields to borrow from the law to align AI and to improve AI for responsible integration into law.
